# Effects of the Application of an Oxygen-Enriched Oil-Based Dressing (NovoX^®^-Drop) After Extraction of Impacted Lower Third Molars: A Randomized Controlled Study

**DOI:** 10.3390/jcm14144986

**Published:** 2025-07-15

**Authors:** Valeria Mitro, Francesco Giovacchini, Massimiliano Gilli, Gabriele Monarchi, Angela Rosa Caso, Antonio Bimonte, Guido Lombardo, Antonio Tullio

**Affiliations:** 1 Department of Maxillo-Facial Surgery, Hospital of Perugia, Sant’Andrea delle Fratte, 06132 Perugia, Italy; f.giovacchini@ospedale.perugia.it (F.G.); massimilianogilli0@gmail.com (M.G.); 2 Department of Medicine, Section of Maxillo-Facial Surgery, University of Siena, Viale Bracci, 53100 Siena, Italy; gabriele.monarchi@gmail.com (G.M.); angelarosa.caso@ospedale.perugia.it (A.R.C.); 3 Department of Surgery and Biomedical Sciences, Unit of Paediatric Dentistry, University of Perugia, Piazzale Gambuli 1, 06129 Perugia, Italy; guido.lombardo@unipg.it; 4 Department of Surgery and Biomedical Sciences, Section of Maxillo-Facial Surgery, University of Perugia, Piazzale Gambuli 1, 06129 Perugia, Italy; antonio.tullio@unipg.it

**Keywords:** extraction, third molar, impacted, oxygen-enriched oil

## Abstract

**Objective:** Lower third impacted molar extraction, despite being a routinary procedure for oral and maxillo-facial surgeons, may often result in a significantly negative impact in patient’s post-operatory quality of life. Among others, treatments based on oxygen-enriched oils have been shown to provide valuable therapeutic benefits in promoting wound healing, and therefore improving the immediate post-operatory symptomatology. The aim of this triple-blinded randomized controlled study is to supplement the existing evidence in the scientific literature by assessing the effectiveness of NovoX^®^-Drop (Moss S.p.A., Lesa, Novara), a specific type of oxygen enriched oil-based device in reducing pain and inflammatory stimulus of post-surgical wounds following the extraction of lower third impacted molars. **Materials and methods:** Seventy-one patients undergoing surgical extraction of a single lower third impacted molar were randomly assigned to receive either NovoX^®^-Drop (Group A) or a glycerin-based gel (Group B). Additionally, both patient groups followed the same standard therapy with amoxicillin-clavulanic acid and ibuprofen. Data were collected preoperative (T0) and after three (T3) and seven (T7) days postoperative in order to assess the following outcomes: mean visual analogue scale (VAS) score during the seven days protocol treatment, total duration of nonsteroidal anti-inflammatory drug (NSAID) usage, trismus (maximum mouth opening) and facial oedema. **Results:** Group A (treatment group) reported significatively lower pain levels at T7 compared to group B (average VAS value during the week: Group A: 3.57 ± 0.39 cm; Group B: 4.47 ± 0.40 cm; *p*-value = 0.0014) despite a significatively shorter period of NSAID usage (average NSAID usage duration: Group A: 2.43 ± 0.38 days; Group B: 3.38 ± 0.44 days; *p*-value = 0.00001). Therefore, trismus seems to be better controlled in group A, although the difference between the groups did not reach the threshold for statistical significance. **Conclusions:** The results of this study suggest that application of NovoX^®^-Drop is capable of significantly reducing the post-operatory pain as well as NSAID usage, representing a promising and effective option for third impacted molar extraction surgery management.

## 1. Introduction

Lower third impacted molar extractions represent routinary procedures for oral and maxillofacial surgeons; however, they may often exert a significantly negative impact on patients’ quality of life, particularly during the immediate postoperative period.

In our experience, typically reported symptoms include pain, facial swelling, trismus (restricted jaw movement due to an antalgic masticatory muscle contraction) and consequently chewing issues [1,2]. Furthermore, poor oral hygiene during the postoperative period can be associated with higher postoperative pain levels, as it contributes to bacterial plaque deposition on the surgical site, promoting bacterial proliferation, local production of toxins and chemical mediators and acting as a trigger of the pathophysiological mechanisms of inflammation [3].

Commonly used treatments in clinical practice, in order to promote wound healing and symptoms control, aside from antibiotics and analgesic drugs, are cryotherapy, chlorhexidine-based gels or mouthwashes, hyaluronic acid-based gels or sprays, platelet-rich plasma and oxygen-enriched oils [1,2,4,5,6,7,8].

Among these supporting treatments, oxygen-enriched oils have been shown to offer several benefits by creating a local microenvironment capable of modulating the local inflammation, and therefore the range of symptoms experienced by patients, improving the postoperative outcomes. The regulation of the inflammatory processes is largely mediated by oxygen and its products. Oxygen availability is strictly related to tissue repair mechanisms, playing a central role in phenomena such as apoptosis, phagocytosis, fibroplasia, extracellular matrix deposition and angiogenesis [9,10]. Furthermore, oxygen and reactive oxygen species (ROS), when locally made available mimicking the macrophage’s release, show bacteriostatic and bactericidal properties, particularly against anaerobes and pyogenic bacteria, as well as against obligate aerobes, which are known to be sensitive to high oxygen levels [11,12]. The effectiveness of oxygen-enriched oils in promoting healing of different types of tissue lesions is widely described in literature [9,12,13,14]. In addition, the use of gel-like formulas can increase the bioavailability of oxygen and reactive oxygen species, by prolonging the application time, similarly to what has been shown for chlorhexidine based-gels, capable of significatively reducing post-extraction alveolar osteitis compared to chlorhexidine rinse [1].

The purpose of the study is to supplement the existing evidence in the scientific literature by assessing the effectiveness of NovoX^®^-Drop (Moss S.p.A, Lesa, Novara), a specific type of oxygen-enriched oil-based medical device, when used in addition to standard anti-inflammatory and antibiotic therapy, in reducing pain and inflammation of post-surgical wounds, as well as trismus and oedema, following the extraction of lower third impacted molars.

## 2. Materials and Methods

### 2.1. Study Design

This study was designed as a randomized controlled triple-blind clinical trial.

### 2.2. Notes on the Medical Device Under Investigations and Comparator Product

NovoX^®^-Drop (Moss S.p.A., Lesa, Novara) is a CE-marked Class IIb medical device composed of 97% oxygen-enriched olive oil with 2% peppermint essential oil and 1% stevia extract as palatability enhancers. The formulation contains no human or animal-derived components. The device is commercially available in single-use syringes (1 mL or 5 mL) for ease of application. The selected comparator product consists of a viscous, colorless, and transparent glycerin-based gel that contains no pharmacologically active substances.

### 2.3. Study Population

The sample was drawn from a population of patients scheduled for the surgical extraction of a lower third impacted molar, after being examined at the Maxillofacial Surgery department of Ospedale Santa Maria della Misericordia in Perugia, Italy. Participant recruitment was carried out among all patients who were seen from 1 May 2024, until the predetermined sample size deemed adequate for this analysis was reached. Before being enrolled in the study, each participant was asked to sign an informed consent form after a researcher clearly explained to them the purpose and procedures of the study, including all its phases. A total of seventy-one patients were enlisted.

### 2.4. Elegibility Criteria

Only patients ≥ 18 years old without any gender distinction, capable of understanding Italian language, were selected. To ensure cohort homogeneity, only patients with a fully mucosal or bony third molar (with fully formed roots) impaction were included, whereas multiple extractions were excluded. Third molars positions were described using Pell–Gregory classifications [15].

Patients with systemic conditions such as history of stroke or myocardial infarction within the past six months, uncontrolled hypertension, kidney or hepatic failure, psychiatric or neurological disorders, including epilepsy, pregnancy or breastfeeding, were not considered suitable for a surgical procedure, and therefore excluded from the study. Additionally, patients presenting with pain, clinical signs of local inflammatory conditions (e.g., periodontitis, local odontogenic abscesses), radiographic evidence of lesions associated with impacted molars (e.g., odontogenic cysts) or, furthermore, participants with anamnestic medical conditions or assuming pharmacological treatments that could compromise post-surgical wound healing (e.g., diabetes mellitus, active bisphosphonates or monoclonal antibodies treatments, immunosuppressive states and primary bone pathologies involving the mandible such as severe osteoporosis or osteonecrosis of the jaw) were excluded. Regular use of antibiotics or anti-inflammatory medications was also considered exclusionary. Lastly, patients with a known or suspected allergy to any of the drugs included in the therapeutic protocol were excluded too.

### 2.5. Treatment Protocol

After a first preliminary visit, patients who met pre-established inclusion criteria, were sequentially added in a surgical waiting list. Each patient on the waiting list was associated with a numerical code in ascending order and linked to an opaque envelope labeled with the same sequential number. Inside each envelope, a card labeled with either the letter ‘A’ or ‘B’ was randomly placed using Microsoft Excel 2024 as a randomization program. Each numbered envelope was sealed, and the numerical sequence was strictly maintained until the completion of all surgical procedures included in the study. Syringes were supplied by the manufacturer in a sealed pouch, labeled as either “A” or “B”. To ensure blinding to both the patient and the healthcare personnel, only the manufacturer was aware of the product associated with each group. Moreover, none of the administering physicians, the patient nor the researcher responsible for evaluating the surgical wound could distinguish between the two product types, based on the external characteristics of the syringes and on the general properties of the products. Patients assigned to group A, received the product from a syringe labeled “A”, while patients assigned to group B received the product from a syringe labeled “B”. The correspondence between the letters (A or B) and the product types (NovoX^®^-Drop or glycerin-based gel) was disclosed to the research team only after both the collection and the statistical analysis of all clinical data were finalized. Based on the described methodology, the study was therefore classified as triple-blinded.

Both patient groups received the exact same post-operatory instructions: a seven-day soft and lukewarm diet, accurate oral hygiene (brushing teeth three times daily), a seven-day antibiotic prophylaxis regimen with amoxicillin-clavulanic acid 1 g per three times a day and an analgesic regimen with ibuprofen 600 mg per maximum three times a day as needed. Each patient was instructed to apply the gel product from the provided syringe on the surgical wound site with a finger after the daily oral hygiene three times a day for seven days taking care not to eat or drink for the following 30 min. To maximize patient compliance, the application procedure was explained both verbally and through practical demonstration during the baseline and follow-up visits, ensuring that each patient was able to correctly replicate the procedure. In our assessment, all patients completed the treatment cycle correctly; however, due to the self-administered nature of the product application at home, it is not possible to guarantee that every application was performed correctly.

### 2.6. Surgical Technique

A troncular local anesthesia of the inferior alveolar and buccal nerves was selected. The mucoperiosteal access flap was chosen taking into account the position, the orientation and the depth of impaction of the third molar. After performing the subperiosteal dissection an ostectomy was carried out to create a favorable leverage point for the periodontal ligament luxation and to provide enough space for the subsequent odontotomy maneuvers. After the extraction, a final revision of the alveolus was performed ensuring the removal of the follicular sac and through abundant irrigation with a sterile saline solution. Resorbable sutures (3/0) were used to close the surgical site.

### 2.7. Outcomes

The study focused on three main postoperative outcomes: (1) oedema, meant as surgical site homolateral cheek swelling grade; (2) trismus, meant as mouth opening reduction following antalgic contraction of masticatory muscles; (3) post-operatory referred pain levels.

Data were collected preoperative (T0) and after three (T3) and seven (T7) days postoperative.

Oedema and facial swelling were measured as the arithmetical mean between the distance, in centimeters, from the corner of the mouth to the attachment of the earlobe and the distance, in centimeters, from the lateral eye canthus and the angle of the mandible. Distances were measured using a flexible instrument capable of remaining in contact with curvatures of the bulged cheek.

Trismus was evaluated by measuring the distance in centimeters, during a maximal mouth opening, from the mesial corner of the upper right central incisor (element 1.1) to the mesial corner of the lower right central incisor (element 4.1), using a specific caliber. The measurement was taken twice, and the average opening value was calculated.

For post-operatory pain level assessment, both objective and subjective parameters were considered. The total duration (in days) of the non-steroidal anti-inflammatory drug (NSAID) treatment was used as the objective parameter, whereas a visual analogue scale (VAS) of 10 cm was used as the subjective parameter. Patients were required to self-assess the average pain level experienced during the entire week, seven days after the procedure was performed [16,17].

### 2.8. Statistical Analysis

Power analysis was performed considering a statistical power of 80%, taking into account an estimated 5% dropout rate the required sample size to ensure adequate statistical power is 71 participants. Kolmogorov–Smirnov analysis was used to assess the normal distribution of data, for all the measured outcomes. Every collected data resulted normally distributed. Statistical significance of normally distributed data was tested using Student’s *t*-test. Statistical significance threshold was set at *p*-value < 0.05. Cohen’s d formula was used to calculate the effect size of pain assessment, trismus and facial swelling parameters between the two groups.

## 3. Results

A total of 71 patients were included in the study and randomly assigned to two different groups: group A (treatment group) with a total of 35 participants (49.30%) and group B (control group) with a total of 36 participants (50.70%) (Table 1).

### 3.1. Baseline Groups Description

Of the 71 patients selected, 43 were female (60.56%) and 28 were male (39.44%). Group A included 22 females (62.86%) and 13 males (37.14%), while group B included 21 females (58.33%) and 15 males (41.67%).

The mean age of participants was comparable between the two groups with no statistically significant differences (Group A: 32.54 ± 4.60 years; Group B:34.41 ± 5.11 years; *p*-value = 0.2978).

Regarding the smoking status, 13 participants in group A (37.14%) and 13 in group B (36.11%) were active smokers.

The average duration of the surgical procedure was slightly longer in group B (51.97 ± 3.22 min) compared to group A (45.46 ± 2.98 min), and this difference resulted statistically significant (*p*-value = 0.0024).

According to Pell–Gregory classification in group A 8 IA, 6 IIA, 0 IIIA, 2 IB, 11 IIB, 1 IIIB, 1 IC, 4 IIC and 2 IIIC were included, whereas in group B 5 IA, 5 IIA, 1 IIIA, 1 IB, 6 IIB, 5 IIIB, 2 IC, 6 IIC and 5 IIIC were included [15] (Table 1).

These data suggest a high level of homogeneity between the two groups at baseline.

### 3.2. Facial Swelling Measurements

Facial swelling was measured preoperative and after three and seven days postoperative:

- T0: Group A: 11.15 ± 0.25 cm; Group B: 11.13 ± 0.24 cm; *p*-value = 0.4517; effect size = 0.02
- T3: Group A: 11.73 ± 0.25 cm; Group B: 11.72 ± 0.23 cm; *p*-value = 0.4634; effect size = 0.01
- T7: Group A: 11.35 ± 0.26 cm; Group B: 11.22 ± 0.24 cm; *p*-value = 0.3847; effect size = 0.12

**Figure 1:**
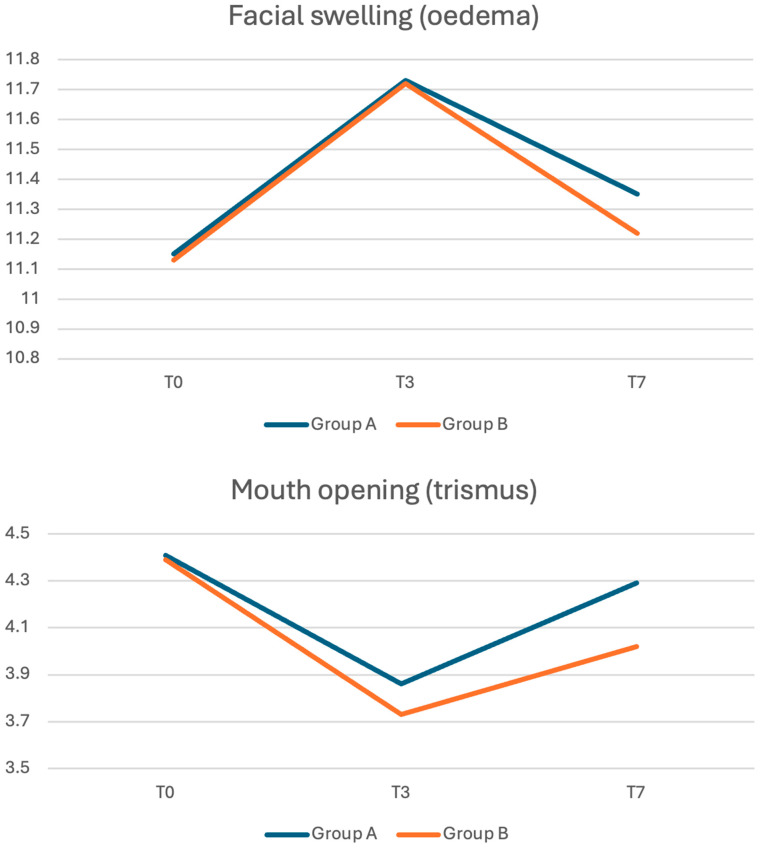
Graphical representations of facial swelling and mouth opening (expressed both in centimeters) before procedure and three and seven days after procedure in group A (blue) and group B (orange) respectively.

No statistically significant differences were measured between the two groups preoperative nor postoperative (Figure 1 and Table 1).

### 3.3. Mouth Opening Measurements

Maximum mouth opening was measured preoperative and three and seven days postoperative:

- T0: Group A: 4.41 ± 0.14 cm; Group B: 4.39 ± 0.12 cm; *p*-value = 0.3996; effect size = 0.05
- T3: Group A: 3.86 ± 0.15 cm; Group B: 3.73 ± o,15 cm; *p*-value = 0.3244; effect size = 0.20
- T7: Group A: 4.29 ± 0.17 cm; Group B: 4.02 ± 0.16 cm; *p*-value = 0.1049; effect size = 0.51

No statistically significant differences were measured between the two groups; nevertheless, the effect size tends to increase over time (Figure 1 and Table 1).

### 3.4. Pain Assessment

Pain assessment was only performed at seven days postoperative:

- Average VAS value during the week: Group A: 3.57 ± 0.39 cm; Group B: 4.47 ± 0.40 cm; *p*-value = 0.0014; effect size = 0.69
- Average NSAID use duration: Group A: 2.43 ± 0.38 days; Group B: 3.38 ± 0.44 days; *p*-value = 0.00001; effect size = 0.68

**Figure 2:**
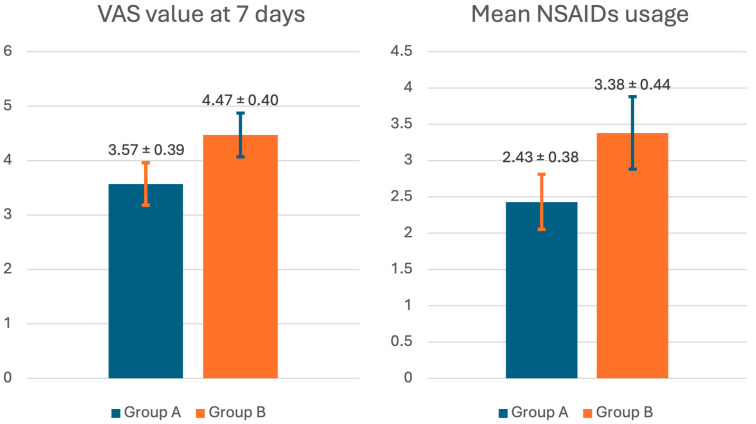
Graphical representations of pain evaluated at seven days after procedure using VAS (expressed in centimeters) and mean usage in days of NSAIDs (expressed in days) in group A (blue) and group B (orange), respectively.

These findings demonstrate a statistically significant difference in both objective (analgesic consumption) and subjective (VAS) pain measures, with group A experiencing lower pain levels despite an inferior NSAID requirement. Furthermore, the effect size can be considered as large (Figure 2 and Table 1).

### 3.5. Adverse Effects

No adverse effects have been reported during the study period.

**Table 1:** Summary of baseline and follow-up collected data for groups A, B, and the total study population. Data, when applicable, are presented as mean values with corresponding 95% confidence intervals. Age is reported in years, procedure duration is measured in minutes, facial swelling, and mouth opening are measured in centimeters (cm,) mean visual analogue scale (VAS) is also expressed in centimeters (cm) and mean non-steroidal anti-inflammatory drugs (NSAIDs) is reported in days. Standard deviation (SD) is provided where applicable. *p*-values and effect size reflect comparisons between Group A and Group B (when not applicable *p*-value is reported as NA).

** Parameters **	** Total **	** Group A **	** Group B **	***p*-Value **	** Effect Size **
** Baseline group description **					
** Participants **	71 (100%)	35 (49.30%)	36 (50.70%)	NA	NA
** Age **	33.49 ± 3.43 (SD 14.74)	32.54 ± 4.60 (SD 13.90)	34.41 ± 5.11 (SD 15.64)	0.2978	NA
** Gender **					
M	28 (39.44%)	13 (37.14%)	15 (41.67%)	NA	NA
F	43 (60.56%)	22 (62.86%)	21 (58.33%)	NA	NA
** Smoking status **					
Smoking	26 (36.62%)	13 (37.14%)	13 (36.11%)	NA	NA
Nonsmoking	45 (63.38%)	22 (62.86%)	23 (63.89%)	NA	NA
** Dental class **					
IA	13 (18.31%)	8 (22.86%)	5 (13.89%)	NA	NA
IIA	11 (15.50%)	6 (17.14%)	5 (13.89%)	NA	NA
IIIA	1 (1.41%)	0 (0%)	1 (2.78%)	NA	NA
IB	3 (4.23%)	2 (5.71%)	1 (2.78%)	NA	NA
IIB	17 (23.94%)	11 (31.42%)	6 (16.67%)	NA	NA
IIIB	6 (8.45%)	1 (2.85%)	5 (13.89%)	NA	NA
IC	3 (4.43%)	1 (2.85%)	2 (5.56%)	NA	NA
IIC	10 (14.08%)	4 (11.43%)	6 (16.67%)	NA	NA
IIIC	7 (9.86%)	2 (5.71%)	5 (13.89%)	NA	NA
** Procedure duration **	48.76 ± 2.30 (SD 9.92)	45.46 ± 2.98 (SD 8.98)	51.97 ± 3.22 (SD 9.85)	0.0024	NA
** Facial swelling measurements **					
** T0 **	11.13 ± 0.17 (SD 1.06)	11.15 ± 0.25 (SD 1.07)	11.13 ± 0.24 (SD 1.06)	0.4517	0.02
** T3 **	11.72 ± 0.17 (SD 1.02)	11.73 ± 0.25 (SD 1.06)	11.72 ± 0.23 (SD 0.99)	0.4634	0.01
** T7 **	11.28 ± 0.18 (SD 1.07)	11.35 ± 0.26 (SD 1.10)	11.22 ± 0.24 (SD 1.05)	0.3847	0.12
** Mouth opening measurements **					
** T0 **	4.40 ± 0.09 (SD 0.39)	4.41 ± 0.14 (SD 0.41)	4.39 ± 0.12 (SD 0.36)	0.3996	0.05
** T3 **	3.79 ± 0.11 (SD 0.45)	3.86 ± 0.15 (SD 0.45)	3.73 ± 0.15 (SD 0.45)	0.3244	0.20
** T7 **	4.15 ± 0.12 (SD 0.51)	4.29 ± 0.17 (SD 0.50)	4.02 ± 0.16 (SD 0.49)	0.1049	0.51
** Pain assessment **					
** Mean VAS **	4.03 ± 0.30 (SD 1.30)	3.57 ± 0.39 (SD 1.19)	4.47 ± 0.40 (SD 1.25)	0.0014	0.69
** Mean NSAID usage **	3.11 ± 0.33 (SD 1.40)	2.43 ± 0.38 (SD 1.14)	3.38 ± 0.44 (SD 1.33)	0.00001	0.68

## 4. Discussion

Oxygen-enriched oils are medical devices in gel-like form consisting of an oil-based matrix enriched with oxygen, capable of continuously releasing microquantities of reactive oxygen species (ROS). These devices are largely used for the treatment of cutaneous lesions and fistulas with remarkable effects in terms of wound healing and bacterial flora control [9,13,14,18]. Despite an extensive body of research on the effectiveness of these dressings in treating cutaneous wounds, a lower number of studies have focused on their application on intra-oral lesions. In vitro studies on clinical samples report that oxygen-enriched oils can effectively limit bacterial and fungal flora growth with comparable effects to those of chlorhexidine and iodopovidone against *Staphylococcus aureus* and *Porphyromonas gingivalis* without measurable cytotoxic effects on fibroblasts and keratinocytes [19,20]. These results have not been subject to a further clinical confirmation through in vivo randomized studies yet. Furthermore, literature data show that the use of topic gel-based or gel-like formulations may increase the application time of the treatment product enhancing the antibacterial effect compared to other formulations such as mouthwash [1]. For these reasons oxygen-enriched oils may represent a valid alternative to other oral wound treatments such as chlorhexidine-based gel or mouthwash avoiding largely known effects like dysgeusia, teeth spotting or oral mucosa irritation. Even though a consistent number of studies demonstrated the effectiveness of chlorhexidine and hyaluronic acid in the treatment of third molar extraction wounds [1,2,4], only a limited body of evidence is available, supporting the need for supplementary research, involving large patient populations, on the use of oxygen-enriched oil-based devices for oral mucosa lesions. This gap in literature led us to design the current study, with a view to assess the actual effectiveness of the above-described treatment specifically for post-third molar impacted extraction wounds. As can be appreciated from the statistical analysis, NovoX^®^-Drop is particularly effective in controlling painful symptomatology. In fact, patients treated with topic oxygen-enriched gels, 7 days after the procedure, experienced less pain compared with non-treated patients, despite the lower usage of painkillers. Both parameters showed a large effect size demonstrating the statistical strength of the effect of the treatment on the painful symptomatology control. In addition, even though in our study the results could not confirm a statistically significant difference, the data seem to suggest that the treatment can positively affect the development of trismus especially at seven days from the procedure. In fact, effect size tends to increase over time until a maximum value of 0.51 showing a medium statistical strength of the measured effect. While post-operative symptomatology, especially in oral surgical procedures, is produced by the constant inflammatory stimulus, oxygen-enriched oils help controlling inflammation by creating an unfavorable substrate for bacterial proliferation and promoting a reparatory response. Taking into account the effectiveness in reducing pain symptoms, the absence of reported side effects and the relative ease of self-application of the product the cost-benefit balance can be considered as positive. Nevertheless, some patients reported a poor palatability of the product after intraoral application as a negative aspect.

### Limits of the Study

This study presents several limitations that need to be acknowledged. Although the sample size was sufficient to achieve a statistical power of 80%, it remains relatively small when conducting a stratified analyses based on smoking status, gender and age. Additionally, the lack of a statistically significant difference in trismus and oedema between the two groups may reflect both a true absence of effect of the treatment on these specific symptoms or an insufficient sample size to detect such differences, highlighting the need for larger-scale studies.

Despite the use of standardized methods for assessing trismus and facial swelling, these kinds of measures inevitably remain partially operator-dependent and challenging to fully standardize in different clinicians, potentially inducing a variability in the obtained results. Another limitation is represented by the difficulty in fully monitoring patients’ application modalities of the product, despite the detailed verbal and practical instructions provided at each visit.

Moreover, the short follow-up period excludes the evaluation of long-term effects of the product itself.

Finally, the procedure duration resulted slightly longer in the control group. This finding may potentially have influenced the patient experience of the procedure and, consequently, the overall outcomes.

## 5. Conclusions

In our pilot study, authors explored the potential of the product in reducing the average use of NSAIDs following the surgical extraction of lower third impacted molars [21]. In the current research, on broader population, similar trends were observed, and data suggest that the treatment may also have a positive effect on pain perception. However, these findings should be interpreted with caution. Further research involving larger populations is needed to validate the results and to better explore the true effectiveness on parameters such as trismus resolution. Nevertheless, oxygen-enriched oil products may represent a promising option for managing postoperative symptoms in third molar extraction procedures.

## Data Availability

The raw data supporting the conclusions of this article will be made available by the authors on request.
